# Primary adenocarcinoma of the thymus: an immunohistochemical and molecular study with review of the literature

**DOI:** 10.1186/1472-6890-13-17

**Published:** 2013-05-31

**Authors:** Maryam Maghbool, Mani Ramzi, Inga Nagel, Pablo Bejarano, Reiner Siebert, Abolfazl Saeedzadeh, Yahya Daneshbod

**Affiliations:** 1 Department of Molecular Pathology, Dr Daneshbod Pathology Laboratory, Shiraz, Iran; 2 Department of Hematology- Oncology, Shiraz University of Medical Sciences, Shiraz, Iran; 3 Institute of Human Genetics, Christian-Albrechts University Kiel and University Hospital Schleswig-Holstein, Campus Kiel, Kiel, Germany; 4 Department of Pathology, JMH/University of Miami School of Medicine, Miami, USA

**Keywords:** Thymus gland, Primary adenocarcinoma, Intestinal differentiation, Thymic carcinoma, Thymoma, Molecular study, CGH array, Immunohistochemical study, Literature review

## Abstract

**Background:**

Primary adenocarcinoma of thymus is extremely rare.

**Case presentation:**

This is a case of primary adenocarcinoma with intestinal differentiation and focal mucin production in the thymus. Thymic cyst was associated with this tumor. Intestinal differentiation was confirmed by immunohistochemical stain with positivity for CDX-2, CK20, villin, MOC31 and focal positivity of CK7. Array comperative genomic hybridization (CGH) analysis showed a complex pattern of chromosomal imbalances including homozygous deletion at the *HLA* locus in chromosomal region 6p21.32.

**Conclusion:**

This rare tumor shows a similar genetic aberration with other studied thymic epithelial tumors.

## Background

Primary thymic carcinomas are very rare tumors. The most common histologic subtypes are squamous cell, adenosquamous/mucoepidermoid, basal cell, large cell undifferentiated, adenocarcinoma, carcinoma with adenoid cystic carcinoma-like features, lymphoepithelioma-like, clear cell, and sarcomatoid carcinomas [1]. Primary thymic adenocarcinoma was first reported in 1989 [2] but has not been accepted as a valid histologic subtype until 1997 [3]. These are uncommon neoplasms. Papillary adenocarcinoma and mucinous adenocarcinoma are the most common variants [1,4-6]. Therefore, before a diagnosis of a de novo thymic adenocarcinoma, other possible diagnoses such as: metastatic carcinoma, thymoma and adenocarcinoma arising in a mediastinalteratoma should be excluded [1,7]. There is little genetic data for thymic carcinomas other than squamous cell carcinomas [8], so we applied a genetic study by array comperative genomic hybridization (CGH) analysis on this case.

## Case presentation

**Figure 1:**
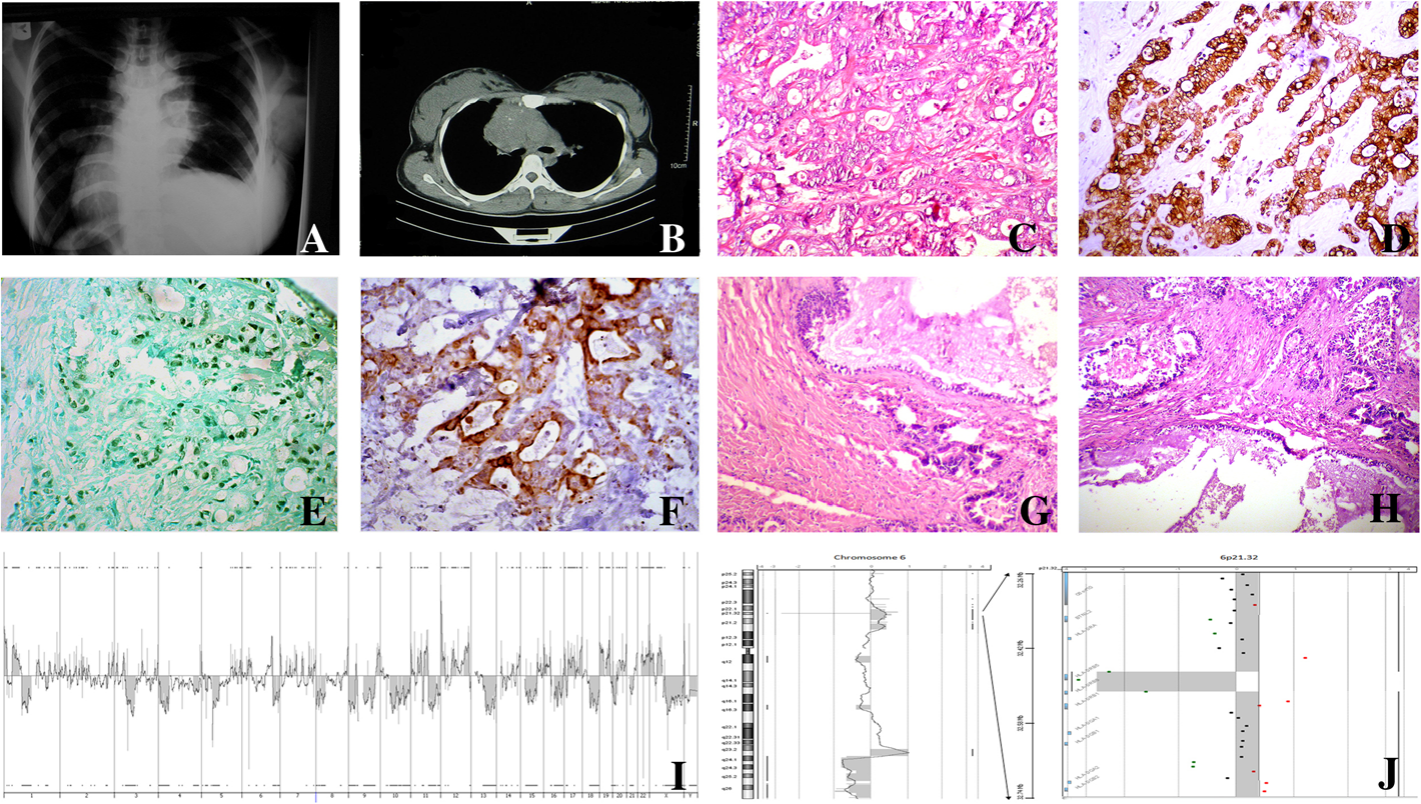
**Representative images of radiology, histology, immunostaining as well as results of array-comparative genomic hybridization (array-CGH). A**: Chest X ray showed mediastinal widening. **B**: chest CT scan revealed anterior mediastinal mass that invade pericardium. **C**: tumoral sheets and glandular structures in desmoplasticstroma (H&E stain, × 400). **D**: Malignant glands showed diffuse membranous positivity for CD5. (Immunoperoxidase). **E**: nuclear positivity for CDX-2 in malignant glands (Immunoperoxidase). **F**: Malignant glands showed diffuse positivity for CK20 (Immunoperoxidase). **G**: benign columnar linningthymic cyst adjacent to neoplastic glands (H&E stain, × 200). **H**: neoplastic glands arising in the vicinity of thymic cyst (H&E stain, × 200). **I**: Array-CGH-results displayed in a whole genome view showed deletion of chromosome 6. **J**: Homozygously deleted region around the HLA-DRB5-locus in chromosomal region 6p21.32.

A 28 year old woman presented with neck and right upper extremity pain accompanied by dyspnea of two years duration. Chest X- ray revealed mediastinal widening (Figure 1A). Chest computed tomography (CT) scan (Figure 1B) showed an anterior mediastinal mass invading in pericardium without extramediastinal extension. An initial clinical impression of a mediastinal germ cell tumor was considered but serum tumor markers such as alpha-feto protein (AFP), Beta-human chorionic gonadotropin (β-hCG), carcinoembryonic antigen (CEA) and CA-125 were normal. CA19-9 level was 2420 U/ml (normal: 0.37U/ml). A mediastinal biopsy was done and showed histologic features of adenocarcinoma. Endoscopy, colonoscopy, abdominopelvicsonography and imaging studies were negative for primary origin. The patient underwent mid-sternotomy. The mass was resected. The patient received chemotherapy (GEMOX) and radiotherapy with two recurrences in 2 years follow up. She is doing well and free of tumor after 6 months.

### Pathologic findings

The mediastinal biopsy showed a moderately differentiated adenocarcinoma with a desmoplastic and necrotic stroma (Figure 1C). Scattered small mucinous lakes were noted in the stroma. The stroma also showed foci of calcification and occasional psammoma bodies. Intracytoplasmic and stromal positive reactions were seen on mucicarmin stain. The excised tumor showed similar morphological and immunohistochemical characteristics. In one of the sections, a unilocunar large cyst with columnar epithelium, in vicinity of the tumor was also identified (Figure 1G, H). Small foci of remnant of thymic tissue were identified. Immunohistochemical (IHC) study showed positivity for CK, EMA, CK20 (Figure 1D), CDX-2 (Figure 1E), CK7, CD5 (Figure 1F), villin, HMWK(focal) and MOC31 but ER, PR, TTF-1, GCDFP-15, P63, CD30, PLAP, chromogranin, calretinin and CD117 were all negative. Commercial sources, clones, and working dilutions of antibodies used in the study are listed in Table 1. Extensive histologic and immunohistochemical study of the whole specimen showed no evidence of teratomatous or germ cell elements.

**Table 1:** Comercial sources, clones, and working dilutions of antibodies used in this case

**Antibody**	**Dilution**	**Clone**	**Vendor**
HMWK	Ready to use	34 βE12	Novocastra, New Castle, UK
EMA	Ready to use	GP1.4	Novocastra, New Castle, UK
CK	Ready to use	AE1/AE3	Novocastra, New Castle, UK
CK20	Ready to use	PW31	Novocastra, New Castle, UK
CDX-2	1: 100	AMT28	Novocastra, New Castle, UK
CK7	Ready to use	RN7	Novocastra, New Castle, UK
CD5	Ready to use	4C7	Novocastra, New Castle, UK
Villin	1: 200	CWWB1	Novocastra, New Castle, UK
MOC31	1: 50	MOC31	Novocastra, New Castle, UK
ER	Ready to use	6 F11	Novocastra, New Castle, UK
PR	Ready to use	16	Novocastra, New Castle, UK
TTF-1	1: 200	SPT24	Novocastra, New Castle, UK
GCDFP-15	Ready to use	23A3	Novocastra, New Castle, UK
P63	1: 25	7JUL	Novocastra, New Castle, UK
CD30	Ready to use	JCM182	Novocastra, New Castle, UK
PLAP	Ready to use	8A9	Novocastra, New Castle, UK
Chromogranin	Ready to use	5H7	Novocastra, New Castle, UK
CD117	Ready to use	T595	Novocastra, New Castle, UK
Calretinin	1: 200	CAL6	Novocastra, New Castle, UK

### Genetic abberations in formalin-fixed paraffin-embedded tissue

Array CGH on this case was performed using the Human Genome Microarray 180A platform (Agilent, Santa Clara, USA). The experimental procedures were performed according to the protocols provided by the manufacturers. Slides were scanned with the G2565CA Microarray Scanner (Agilent) at a scan resolution of 5 μm. Signal intensities from the generated images were measured and evaluated with Feature Extraction 10.10.11 and Agilent Genomic Standard Workbench Edition 6.5.0.58 (AGW6.5) software (Agilent) applying the Aberration Detection Method-2 (ADM-2) algorithm with a threshold of 6.0. It showed a complex karyotype with multiple gains and losses. Part of the *HLA-DR5* locus in chromosomal region 6p21.32 seemed to be homozygously deleted (Figure 1I, J).

### Consent

“Written informed consent was obtained from the patient for publication of this case report and any accompanying images. A copy of the written consent is available for review by the Editor-in-Chief of this journal.”

## Discussion

Metastatic neoplasms to the mediastinum account for most of epithelial cell neoplasms. The second most common tumors are thymoma and thymic carcinoma. Primary adenocarcinoma is very rare in mediastinum, so before considering this diagnostic entity, most prevalent tumors such as metastatic adenocarcinomas, germ cell tumors and malignant teratomas must be ruled out [1,3-5,7,8]. Through clinical history, imaging studies, absence of extramediastinal tumor and histology, we report a case of primary adenocarcinoma with intestinal differentiation of the thymus with mucin production.

Immunostains are an important tool in the study of mediastinal tumors. For instance CD5 is a leukocyte marker expressed on differentiating thymocytes. It is said to be useful in differentiating thymic from non-thymic carcinomas [4,7,8]. Caution must be given because CD5 is positive in malignant pleural mesothelioma and adenocarcinomas of other (non thymic origin) organs [4,8]. It is also important to exclude tumors derived from the lung and pleura by evaluating TTF-1 and calretinin.

The exact origin of thymic adenocarcinoma is not clear. Glandular differentiation is rarely seen in ultrastructural studies of normal thymic epithelial cells [5]. Also, in the involuted thymus, glandular or tubular structures can be found [7]. Therefore, adenocarcinomas can result from extreme glandular differentiation during tumor progression.

Review of the literature on thymic adenocarcinoma is shown in Tables 2 and 3. Twenty-six cases have been reported. Patients range in age from 15 to 82 years (mean 50 y ±17 y). Male/female ratio is 1.9/1. The most common morphologic subtypes are papillary carcinoma (38%), mucinous adenocarcinoma (34%), conventional adenocarcinoma (0.11%), NOS (0.07%) and papilotubular carcinoma (0.07%). Median age is 52.56 years. Associated findings are thymic cyst in 26% (mostly seen in mucinous subtype), thymoma in 11% (only in papillary subtype) and psammoma bodies in 15% (mostly seen in papillary subtype). Serum tumor markers were increased. CEA in 23% (mostly seen in mucinous subtype), B-HCG in 0.38% (only in conventional subtype) and CA19-9 in 11% (one papillary, one NOS and two mucinous subtypes, including ours). Immunohistochemistry on different subtypes were performed in a limited number of papers and showed positivity in CK7 (7/11), CK20 (6/9), CEA (6/9), Leu M1 (4/5), B-HCG (1/2), CDX-2 (3/4), Muc2 (1/2), Muc5 (2/2), CD5 (8/12), P63 (1/2), CA-19-9 (3/3), CAM5.2 (2/2), CK5,6 (1/2), P53 (2/2), Her2 (1/2) [4-7,9-18].

**Table 2:** Literature review of clinicopathological data of primary thymic adenocarcinomas

**Author**	**Year**	**Sex**	**Age**	**Symptoms**	**Tumor type**	**Site**	**RX**	**Outcome**	**Associated findings**
Moriyama S [2]	1989	F	51	Hyperthyroidism	Papillo-tubular	Right lobe of the thymus	S	No recurrence for 14 years	Multiple thymic cysts
Babu MK [19]	1994	M	50	NA	Conventional	NA		Alive after 4 months	Congenital thymic cyst
Makino Y [6]	1998	M	39	Chest pain	Papillary carcinoma	NA	S,R	Alive after 5 months	NA
Matsuno [5]	1998	M	70	Not mentioned	Papillary carcinoma	Anterior mediastinum	S,R	Recurrence after 8 months	Numerous psammoma bodies
		F	69	Not mentioned	Papillary carcinoma	Left lower pole of the thymus	S	Not mentioned	Thymoma of medullary type
		F	61	Not mentioned	Papillary carcinoma	Substernalmediastinal mass	S,R,C	Died after 7 months	Chronic elephantiasis nostrasverrucosum in skin
		M	57	Not mentioned	Papillary carcinoma	Anterior mediastinum	S,R,C	Alive after 5 years	No
Shimono [20]	2001	M	61	facial edema, general fatigue	Not specified	Anterior mediastinum	S,R,C	Alive after 53 months	No
Zaitlin [21]	2003	F	51	Asymptomatic	Papillary carcinoma	Anterior left mediastinum	S,R	Died after 26 months	Thymic cyst
Choi WW [7]	2003	M	39	Cough&lethargy	Papillary carcinoma	Anterior mediastinum	S,R,C	Alive after 3 years	NA
		M	15	Dry cough	Mucinous carcinoma	Right anterior mediastinum	S,R	Died after 26 months	Thymiccyst&cribriform, carcinomatous gland in thymic medulla
Misao [10]	2004	M	59	Accidental finding in chest x ray	Not specified	Anterior Mediastinum	S,C	Died after 24 months	No
Takahashi F [1]	2004	M	49	Accidental finding in chest x ray	Mucinous carcinoma	Anterior mediastinum	R	Died 11 months after DX	Increased CA19-9, CEA
Seki, ErinaMD [11]	2004	M		Left shoulder pain	Mucinous adenocarcinoma with pleural dissemination	Anterior mediastinum	R.C	Alive in 11 months after surgery.	No
Yoshino M [12]	2005	F	29	Accidental finding in chest x ray	Papillary carcinoma	Anterior mediastinum	S	Not mentioned	Type A thymoma, Psammoma bodies and follicles
PayalKapur [4]	2006	M	41	Not mentioned	Mucinous carcinoma	Anterior mediastinum	R,c	2 recurrence in 3 years	Multiple thymic cysts
ToyomitsuSawai [9]	2006	M	34	Accidental finding in chest x ray	Moderately differentiated adenocarcinoma	Right anterior mediastinum	R,C	Lung metastasis after eight months of surgery	Increased CA19-9, CEA
Seong H. Ra [13]	2007	F	61	Hoarseness, dysphagia, left shoulder pain, and fatigue	Mucinous carcinoma	Superoanterior mediastinum	S,R	Metastatic disease to retroperitoneal lymph nodes at 5 months	Psammoma bodies
		F	82	Shortness of breath, chest tightness, back stiffness, and weight loss of 15 lb	Mucinous carcinoma	Anterior mediastinum	S	Passed away due to surgical complications	Thymiccyst, signetringlike features
Furtado [14]	2008	M	44	Accidental finding in chest x ray	Papillary carcinoma	Pretracheal region	S,R,C	Alive for 24 months	Psammoma bodies
HosakaY [15]	2009	M	36	Accidental finding in chest x ray	Moderately differentiated adenocarcinoma with papillary formation	Right anterior mediastinum	R	Alive in 11 years after surgery	Type AB thymoma
Toshiji I [16]	2009	M	54	Cough, fever, dyspnea,chest pain, facial edema	Poorly differentiated sarcomatoid tumor cells partially composed of papillotubular adenocarcinoma	Anterior mediastinum	R	Passed away 56 days after the initialsymptoms	Slight elevation of sialyl Lewis-x antigen,soluble interleukin-2 receptor
Maeda D [17]	2009	F	52	Bulging of the left parasternal region	Mucinous carcinoma	Anterior mediastinum	S,R,C	Lung metastasis after 7 months	Thymic cyst
		M	38	Chest pain	Mucinous carcinoma	Anterior mediastinum	S,R,C	Died 12 months after diagnosis	No
		M	55	Chest tightness	Mucinous carcinoma	Anterior mediastinum	S,R,C	Died 24 months after diagnosis	No
Yong Joon Ra [18]	2010	M	68	Dyspnea and chest pain on left anteriorchest	Conventional	Anterior mediastinam	Not mentioned	Not mentioned	Slightly increased (β-hCG) level
Current case	2010	F	28	Neck and right upper extremity pain and dyspnea	Mucinous	Anterior mediastinam	S,R,C	Two recurrences in two years follow up	Thymic cyst and psammoma bodies, increased CA19-9

Abbreviations: *NA* not available, *S* Surgery, *R* Radiotherapy, *C* Chemotherapy, *Thy* Thymectomy.

**Table 3:** Clinicopathologic data of the thymic adenocarcinoma reported in literature according to the four major subtypes

**Tumor subtype**	**Total case**	**Male/Female**	**Median age**	**Associated thymic cyst**	**Associated thymoma**	**Associated psammoma body**	**Associated serum tumor markers**			
							**CEA**	**B-HCG**	**AFP**	**CA19-9**
Papilotubular	2	1	52.5	1	0	0				
Conventional	3	3	50.6	1	0	0	1	1		
Papillary	10	1.5	49.5	1	3	3	1			1
NOS	2	2	60	0	0	0	1			1
Mucinous	9	2	50.2	4	0	1	3			1

In our case the adenocarcinoma was associated with a large benign thymic cyst with columnar epithelium. It showed no dysplastic change.

Most of the patients with thymic adenocarcinoma do not have any chief complaint (41%). In symptomatic group, the most common presenting sign is chest pain (0.17%). Other rare signs are cough (0.05%), dyspnea (0.05%), and shoulder pain (0.05%). Weight loss is a rare sign in this tumor (0.05%). Anterior mediastinum is the most common location (79%) followed by right, left, substernal and pretracheal mediastinum (each below%1). Prognosis cannot be accurately evaluated due to low incidence rate of thymic adenocarcinoma. Some patients underwent surgical resection (15 cases), chemotherapy (12 cases) or radiotherapy (19 cases). Clinical outcome showed local recurrences (2 cases), metastatic disease (3 cases) and death due to surgical complication or disease (9 cases). According to Table 3, mucinous has much worse prognosis than papillary carcinoma (p value < 0.05). There are diverse genetic heterogenecity in thymic tumors. Genetic characterization has concentrated on WHO types A, B3, and C that harbors few lymphocytes [22]. No chromosomal gain is seen in type A and AB thymomas [8]. Simultaneous gain of 1q and loss of chromosome 6, and 13q aberrations frequently detected in type B3 thymomas. Loss of heterozygosity (LOH) on chromosome 6 is the most frequent genetic abnormality in thymoma. Recent studies on genetic alterations of thymoma based on (LOH) analyses inferred two different genetic pathways of tumorigenesis of thymoma, and heterogeneous genetic alterations in subtypes of thymoma, were identified by CGH and LOH analyses [22,23]. One of the important findings in the thymic epithelial tumor is frequent and multiple genetic aberrations of chromosome 6 that are found in 77.5 percent of them. There are five hotspots of frequent deletions indicating that several putative tumor suppressor genes on chromosome 6 are involved in the development of thymic epithelial tumor [8,22,23]. Deletion sites such as 6q21, 6q23, and 6q25-27 are well established in thymoma. As Zhou et al. reported; the most frequent LOH was found in the 6q23.3-25.3 chromosomal region and the second hot spot of deletions was located in the 6p21 region containing the major histocompatibility (MHC) classes I and II gene loci [22,24].

In our case, the *HLA*-*DRB5*-locus in chromosomal region 6p21.32 was homozygously deleted, showing similar genetic aberrations with other thymic epithelial tumors.

## Conclusion

This primary adenocarcinoma of the thymus with intestinal differentiation shows homozygous deletion of part of the *HLA*- locus in chromosomal region 6p21.32 confirmed by CGH array. It is associated with a benign thymic cyst. This is the first genetic study on a primary thymic adenocarcinoma (mucinous type), which shows a similar genetic aberration with other thymic epithelial tumors. We suggest adding genetic study to clinicopathologic and imaging workup for confirmation of diagnosis of a primary thymic tumor.

## Competing interests

No conflict of interest is declared, and source of funding is Dr. Daneshbod Laboratory.

## Authors’ contributions

YD, MM, MR and AS were involved in diagnosis, conception, design, acquisition of data, analysis and interpretation of data and were directly involved in drafting and revising the manuscript. IN and RS were participated in the array CGH, analysis and interpretation of data and revising the manuscript. PB reviewed this case and confirmed the diagnosis as well as extensively revised the paper. All authors read and approved the final manuscript.

## Pre-publication history

The pre-publication history for this paper can be accessed here:

http://www.biomedcentral.com/1472-6890/13/17/prepub
